# Molecular characterization of Extended-spectrum β lactamase- producing *E. coli* recovered from community-acquired urinary tract infections in Upper Egypt

**DOI:** 10.1038/s41598-020-59772-z

**Published:** 2020-02-17

**Authors:** Noha A. Hassuna, Ahmed S. Khairalla, Eman M. Farahat, Adel M. Hammad, Medhat Abdel-Fattah

**Affiliations:** 1 Medical Microbiology and Immunology Department. Faculty of Medicine, Minia University, Minia, Egypt; 2 Microbiology and Immunology Department, Faculty of Pharmacy, Beni-Suef University, Beni-Suef, Egypt; 3 Department of Science and Engineering, Red Deer College, Red Deer, Alberta Canada; 4 Microbiology and Botany Department, Faculty of Science, Beni-Suef University, Beni-Suef, Egypt; 5 Microbiology Department, Faculty of Agriculture, Minia University, Minia, Egypt

**Keywords:** Microbiology, Medical research

## Abstract

Treatment of community urinary tract infections (UTIs) caused by extended-spectrum β lactamase (ESBL)- producing *Escherichia coli* (*E. coli*) is more expensive than treating ESBL-negative opposites. Evaluation of the prevalence of ESBL-production among urinary *E. coli* isolates is crucial due to its great impact on the choice of proper antimicrobials. Accordingly, the aim of this work was to detect and characterize ESBL-producing *E. coli* isolated from outpatients with signs of UTIs in Upper Egypt. Urinary *E. coli* isolates were identified by 16S rRNA and their ESBL-production was confirmed by Modified Double Disc Synergy Test (MDDST) and ESBL- CHROMagar media. Isolates were then subjected to Polymerase Chain Reaction (PCR) for new Clermont phylogrouping, ESBL genes detection and CTX-M typing. The study enrolled 583 patients with clinically diagnosed UTIs. Uropathogens were found in 400 urine samples (68.6%) out of which 134 *E. coli* isolates were identified. Among the examined uropathogenic *E. coli* (UPEC), 80 (59.7%) were recognized as ESBL-producers. Greater than half of the ESBL-producers were multi-drug resistant (MDR) (62%). All of them were susceptible to meropenem. Most of the *E. coli* isolates were distributed in 4 phylogenetic groups: B2 = 42 (52.5%), F = 17 (21.25%) and Clade I or II = 10 (12.5%). The predominant gene types were TEM 60 (75%) and CTX-M gene 45 (56.25%). The CTX-M-1 group was the most prevalent (62.2%), including the CTX-M-15 enzyme, followed by the CTX-M-2 group, CTX-M-8 group and CTX-M-9 group. In conclusion, the results present alarming evidence of a serious spread of ESBL genes in Egypt, especially the epidemiological CTX-M 15, with the potential for the dissemination of MDR UPEC strains in the community.

## Introduction

Extended-spectrum β-lactamases (ESBLs) are plasmid-mediated β-lactamases recognized for their ability to hydrolyze 3rd- and 4th-generation cephalosporins (oxyimino-cephalosporin) and monobactams but not cephamycin or carbapenems. Additionally, these enzymes are repressed by *β*-lactamase inhibitors as clavulanic acid and tazobactam. Typically, an ESBL evolved from a narrow spectrum parent, for example from *bla*_TEM-1_, *bla*_TEM-2_, or *bla*_SHV_^1^. Recently, a new class of ESBL genes called *bla*_CTX-M_ have appeared and posed a great burden on the health environment^2^. The worldwide dissemination of *bla*_CTX-M_ producing *E. coli* has been increasing, and are now known to be the main ESBL genes^3^. The first analysis and alignment of the amino acid sequences of the CTX-M variants categorized these enzymes into five clusters: CTX-M-1, CTX-M-2, CTX-M-8, CTX-M-9, and CTX-M-25^4^. Although different informs about CTX-M β-lactamases have been available, updated data about dispersion of CTX-M producing isolates, molecular epidemiology, protein plasticity, evolution and origin of the *bla* genes, influence of antibiotic use, and patients risk factors are still lacking in some regions^5^.

The spread of ESBLs in Enterobacteriaceae has become an ever-increasing problem^6^, with a global rise of ESBL-producing Enterobacteriaceae^7^. One of the most frequently found Enterobacteriaceae harboring ESBL genes is *Escherichia coli* (*E. coli*), where multi-drug resistance (MRD) due to ESBL production is rapidly becoming a threat to the community^8^. Spreading rates of nosocomial ESBL producing *E. coli* are markedly variable, with flat trends in Europe ~15%, and increasing trends in North America from 7.8% in 2010 to 18.3% in 2014^9^. In fact, propagation of MDR and ESBL-producing *E. coli* strains reduces the treatment preferences. It is also mandatory to be informed with the predominant resistant pattern of any region, which could assist in proper antimicrobial therapy^10^.

A recent study done by our group^11^, showed a disturbingly high level of ESBL producers in the urine of asymptomatic pregnant women. Thus, we aimed at evaluating their incidence in patients suffering from UTIs in the community. To the best of our knowledge, there are no previous epidemiological data regarding the phylogenetic grouping of ESBL-producing *E. coli* causing UTIs in Egypt. Accordingly, a phenotypic and a genotypic evaluation of ESBL- producing *E. coli* was carried out, followed by phylogenetic grouping of the obtained isolates.

## Results

### Isolation of *E coli* from urine samples

Out of 583 urine samples obtained from patients suffering from UTIs, 400 isolates were confirmed as positive cultures. 134 of these cultures were confirmed to be *E. coli* (33.5%) (Figure S1).

### Phenotypic detection for esbl production

**Figure 1:**
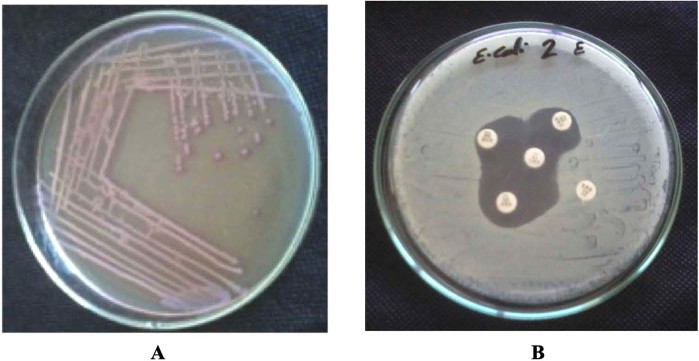
(**A**) *E. coli* on chromogenic agar. (**B**) Detection of ESBL production tested by the Modified Double Disc Synergy Test (MDDST). The test was done by using a disc of amoxicillin-clavulanate (20/10 μg) along with four cephalosporins; cefotaxime, ceftriaxone, cefpodoxime and cefepime.

Out of 134 *E. coli* isolates, 80 (∼60%) were ESBL producers by CHROMagar ESBL screening and 75 (56%) using Modified Double Disc Synergy Test (MDDST) (Fig. 1). There was no statistical significance between CHOROMagar and MDDST regarding detection of ESBL-production (*P* > 0.5; X² = 0.245). Different patterns of synergism of 3^rd^ generation cephalosporin and 4^th^ generation cephalosporin with Amoxicillin Clavulanate using MDDST were observed on testing 80 CHROMAgar ESBL producing *E. coli* isolates (Table 1).

**Table 1:** MDDST for 80 possible ESBL producing *E. coli* isolates:.

Pattern of synergism	Number	Percentage (%)	Cause of resistance
No synergism of 3GC and 4GC with AMC	5	6.25%	Non-ESBLs
Synergism of both 3GC and 4GC with AMC	75	93.75%	ESBLs
Synergism of 3GC only with AMC	0	0%	ESBLs

### Antibiotic susceptibility pattern of ESBL producing *E. coli* isolates

The overall antimicrobial resistance in ESBL producing *E. coli* isolates is summarized in Table 2. The rates of antibiotic resistant *E. coli* were 100% for penicillin group (ampicillin (AMP), piperacillin (PRL), ofloxacin (OFX)), cephems (cefotaxime (CTX), ceftazidime (CAZ), Cefpodoxime (CPD), cefepime (FEP)) and monobactams (aztreonam (ATM)), 82.5% for ampicillin/sulbactam (AMC), 76.25 for sulphamethoxazole-trimethoprim (SXT), 28.75% for Chloramphenicol (C), 23.75% for gentamicin (CN), 13.75% for ciprofloxacin (CIP), and 0% for meropenem (MEM). Most of isolates (>80%) were susceptible to ciprofloxacin, while all the isolates were susceptible to meropenem. About 62% of the isolates were MDR with resistance to one antimicrobial agent in at least 3 different groups.

**Table 2:** Antibiotic susceptibility pattern of ESBL producing *E. coli*.

Sensitivity of isolates	Antimicrobial													
	CPD	ATM	CIP	AMP	CAZ	AMC	MEM	CN	CTX	OFX	C	PRL	SXT	FEP
R	95%	71.25%	13.75%	90%	75%	82.5%	0%	23.75%	97.5%	17.5%	28.75%	47.5%	76.25%	50%
I	3.75%	11.25%	0%	2.5%	11.25%	5%	0%	2.5%	0%	0%	6.25%	32.5%	5%	20%
S	1.25%	17.5%	86.25%	7.5%	13.75%	12.5%	100%	73.75%	2.5%	82.5%	65%	20%	18.75%	30%

R = resistant, I = Intermediate, S = sensitive.

### Phylo-Grouping profile of isolates

**Figure 2:**
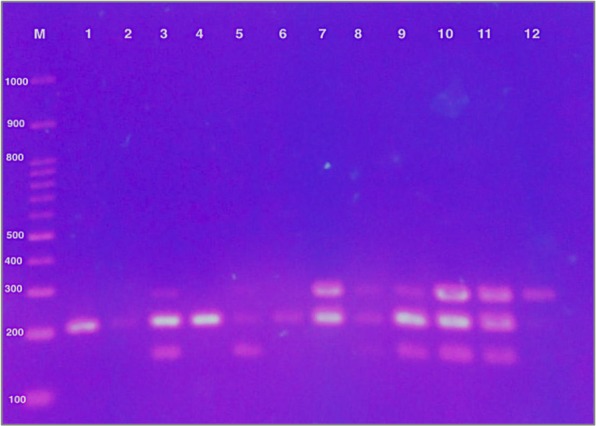
Quadruplex PCR profiles of Clermont phylo-typing method of *E. coli* isolates. Lane M contained 100 bp marker; Lane 3, 7, 8, 9,10 and 11, group B2; Lane 12, group F; Lane 1,2, 4 and 6, Clade I or II; Lane 5, unknown.

Phylogenetic grouping was done according to modified Clermont method^12^. Forty-two isolates (52.5%) were classified into B2 group, 17 isolates (21.25%) in Group F, 10 isolates (12.5%) in Clade I or II, while 11 isolates (13.75%) did not belong to any group (Fig. 2) (Table 3).

**Table 3:** Phylogenetic analysis of 80 *E. coli* isolates (Positive for ESBL genes) Causing urinary tract infection derived from various hospitals in Egypt.

Phylogenetic groups	Genes Combination Profile	Number of isolates	(%) of isolates
A	(arpA*+, chuA-, yjaA-, TspE4.C2-*)	0	0%
B1	(arpA*+, chuA-, yjaA-, TspE4.C2+*)	0	0%
F	(arpA*-, chuA+, yjaA-, TspE4.C2*-)	17	21.25%
B2	(arpA*-, chuA+, yjaA+, TspE4.C2*-)	10	B2 = 42 (52.5%)
B2	(arpA*-, chuA+, yjaA+,TspE4.C2+*)	32	
B2	(arpA*-, chuA+, yjaA-,TspE4.C2+*)	0	
A or C	(arpA*+, chuA-, yjaA+,TspE4.C2*-)	0	0
D or E	(arpA*+, chuA+, yjaA-,TspE4.C2*-)	0	0
D or E	(arpA*+, chuA+, yjaA-,TspE4.C2+*)	0	0
E or clade I	(arpA*+, chuA+, yjaA+, TspE4.C2-*)	0	0
Clade I or II	(arpA*-, chuA-, yjaA+, TspE4.C2-*)	10	12.5%
Clade III, IV or V	(arpA*-, chuA+*(*476*)^*C*^*, yjaA-, TspE4.C2-*)	0	0
Unknown	(arpA*-, chuA-, yjaA-,TspE4.C2+*)	4	Unknown = 11 (13.75%)
Unknown	(arpA*-, chuA-, yjaA+,TspE4.C2+*)	3	
Unknown	(arpA*+, chuA-, yjaA+, TspE4.C2+*)	0	
Unknown	(arpA*+*, chuA*+*, yjaA*+*, TspE4.C2*+*)	0	
Unknown	(arpA*-, chuA-, yjaA-, TspE4.C2-*)	4	

### Molecular detection of ESBL genes

The predominant ESBL gene in this study was *bla*_TEM_, which was found in 60 isolates (75%) (Figure S2). The *bla*_CTX-M_ gene was found in 45 isolates (56.25%), whereas *bla*_SHV_ was found in only 15 isolates (18.75%) (Figure S3 & S4). Most of the isolates (66.25%) showed coexistences of more than one gene, with (28.75%) of ESBL-producing *E. coli* harboring *bla*_TEM_, *bla*_SHV_, and *bla*_CTX−M_. Coexistences of two genes was also observed where *bla*_TEM_ and *bla*_CTX−M_ were detected in (21.25%) of the isolates, *bla*_TEM_ and *bla*_SHV_ in (6.25%) of the isolates, while *bla*_SHV_ and *bla*_CTX−M_ were found in (10%) of the isolates. About 25% of the isolates harbored *bla*_TEM_ alone, and 13.75% had *bla*_CTX-M_ alone. None of the isolates had *bla*_SHV_ alone. Distribution of tested ESBL genes among different groups is found in Table 4.

**Table 4:** Frequency of the different *ESBL* types among ESBL producing *E. coli* isolates of the different phylogenic types.

ESBL-gene	*bla*_TEM_	*bla*_SHV_	*bla*_CTX-M_	*bla*_*C*TX-M_ Sub-types				
				CTX-M-1 (n = 22)	CTX-M-2 *(*n = 12)	CTX-M-8 *(*n = 10)	CTX-M-9 (n = 1*)*	CTX-M-25 (n = 0)
**Phylo-group**								
B2	30	9	37	17	12	7	1	0
F	6	1	2	2	0	0	0	0
Clade I/II	15	3	1	1	0	0	0	0
unknown	9	2	5	2	0	3	0	0
Total of ESBL isolates	60	15	45	22	12	10	1	0

### Characterization of CTX-M-producing *E. coli* clinical isolates

Out of the 45 *bla*_CTX-M_ isolates, Group 1 enzymes were found in 22 isolates (48.9%) (Figure S5 & S6), while CTX-M 15 enzymes were found in 11 isolates (24.4%) (Figure S7). Group 2 enzymes were produced by 12 (26.7%) isolates. The rest of the isolates included: 10 isolates that produced group 8 CTX-M enzymes and one isolate that produced group 9 enzyme. On the other hand, 3 *E. coli* isolates harbored both group 1 and group 8 enzymes. There were no producers of group 25 CTX-M enzymes detected in this study.

## Discussion

Investigating the prevalence of antimicrobial resistance rates is of great importance in both creating strategies for empirical treatment and in evaluating the existing guidelines. The frequencies and types of infections caused by ESBL-producing Enterobacteriaceae have increased dramatically in the past few decades with disparity between different institutions and countries. Since the beginning of the new millennium, *E. coli* has become the most commonly isolated ESBL-producing bacteria worldwide with CTX-M ESBLs being the most frequently isolated types^13^. This upsurge in ESBL-producing *E. coli* adds a great burden to the treatment of community-onset UTIs as such isolates are frequently multidrug-resistant, with increased chance of treatment failure^14–16^. Since there is no comprehensive surveillance of community-acquired UTIs caused by *E. coli* in Egypt, this study was aimed at evaluating the prevalence and the mechanisms underlying their ESBL production. To the best of our knowledge, this is the first article from Upper Egypt to report the characteristics of ESBL-producing *E. coli* from community-onset UTIs.

Out of a total of 134 clinical isolates of *E. coli*, 80 isolates (59.7%) were ESBL positive. This high frequency of ESBLs is comparable to those found in Egypt by Al-Agamy *et al*. and Abdel-Moaty *et al*.^17^, where 52% of the detected isolates were ESBL producers, however, their study did not demonstrate whether the isolates were community or hospital-acquired. On the other-hand, our reported incidence is much higher than that reported earlier in Egypt by Fam *et al*., with a lower prevalence of 17% among community-acquired UTIs^18^, suggesting an increasing trend in the incidence of ESBLs-producing *E. coli* in Egypt. Compared to countries in the same region, our studies were also higher than that found in Lybia (6.7%)^19^ and Emirates (39%)^20^. However, comparable results (67%) were obtained by Zorgani *et al*. (in a different larger-scale study done in Lybia), who reported a high incidence of ESBL-producers among hospital isolates^21^.

Within different ESBLs, CTX-M enzymes are the most predominant in different epidemiological settings, which have outnumbered other ESBL enzymes such as TEM and SHV^22,23^, with more than 172 CTX-M variants reported to date. Al-Agamy *et al*. reported the first detection of CTX-M β-lactamase production by urinary nosocomial *E. coli* isolates in Egypt. They found a high incidence of ESBLs-producing isolates in a single hospital (60.9%)^24^. In this study the predominant gene types were *bla*_TEM_ in 60 isolates (75%) and *bla*_CTX-M_ in 45 isolates (56.25%), while *bla*_SHV_ was found in only 15 isolates (18.75%). The relatively higher frequency of *bla*_CTX-M_ among our community isolates is concurring with the fact that CTX-M ESBLs originate from environmental bacteria unlike TEM- or SHV-ESBLs^25^. These findings are in agreement with previous studies done in Egypt where *bla*_CTX-M_ was prevailing^24,26^.

Interestingly, *bla*_TEM_ was detected in most isolates although it is commonly found in hospital strains, this could probably be due to previous contact with hospitals.

Among *bla*_CTX-M_, *bla*_CTX-M-15_ was the most prevalent (37.8%) in our study, which concurs with various reports demonstrating the extensive worldwide dissemination of *bla*_CTX-M-15_ mediated by clonally related *E. coli* strains^27^.

Regarding the susceptibility profile of ESBLs-producing isolates; all of the isolates were resistant to cephems, which is concurring with previous studies^28,29^.

Our detected resistance rates with ampicillin/sulbactam (AMC) (82.5%) and sulphamethoxazole-trimethoprim (SXT) (76.25%) were higher than that previously reported^26,30,31^ but in agreement with Abdel-Moaty *et al*.^17^.

On the other hand, about 13% of the isolates were resistant to ciprofloxacin using CLSI disk breakpoints^32^, which is much lower than previously reported in Egypt by Abdel-Moaty *et al*.^17^ or in the Middle East region^19,33^. This decreased resistance rate could be due to a better understanding and a wiser use of fluoroquinolones in UTI cases.

In addition, all the isolates were susceptible to meropenem, which is in consensus with a previous study done in the same region on asymptomatic urinary carriers of ESBL-producer strains^11^, who reported that all ESBL producers were sensitive to imipenem (100%).

Alteration in the phylogenetic types are important in identifying novel groups of emerging bacteria that are better recognized as a result of this analysis. Phylogenetic grouping in this study was done according to a modified Clermont method^12^, which was done for the first time on the Egyptian isolates. This new method used modified primers for chuA, yjaA and TspE4.C2, which eradicated some primer mismatches. The most imperative benefit of this method was its power to distinguish strains belonging to phylogroups C, E, F and clade I. In this study most of the isolates were in group B2 (52.5%), followed by group F (21.25%), Clade I or II (12.5%), while 13.75% were of unknown type. The high frequency of phylogenetic group of B2 (52, 5%) is comparable to previous studies^34–36^, where the B2 subgroup was the most common group especially among CTX-M15 strains, as well as phylogroup F, which is closely similar to phylogroup B2^37^.

The presence of Clade I or II isolates (cryptic lineages which are phenotypically similar to *E. coli* but genetically divergent^38,39^) require further investigation as this is the first report for the presence of such clades among extra intestinal isolates in Egypt. Interestingly, cryptic isolates found in this study harbor a variety of ESBLs: TEM, SHV and CTX-M, suggesting a threatening horizontal gene transfer in our community. Environmental spread of ESBL-producing *E. coli* could be attributed to the release of wastewater into rivers^40,41^, where mobile genetic elements are allowed to transfer ESBL-production from environmental bacteria to human and animals^42^.

In conclusion, our data underscores the importance of continuous surveillance of antimicrobial resistance in community *E. coli* isolates and shows the alarming increases of ESBL-production among such strains. Public health efforts should focus on the correct use of antibiotics to limit their dissemination and further investigation of molecular epidemiology of ESBLs in various clinical samples would be promising to obtain a better database for ESBL-producing *E. coli* in Egypt.

## Methods

### Study design

This cross-sectional study was conducted to assess the prevalence and antimicrobial resistance pattern of ESBL-producing *E. coli* isolates from patients with suspected community-onset UTIs during the period of August 2016 to February 2018 from Minia General Hospital, Kidney Hospital, Suzan Mubarak Hospital and Liver Virus Unit in Minia Governorate (located in Upper Egypt). Community-onset infections were defined as infections that have an onset in less than 48 hours of hospital admission or that present in the outpatients’^43^. Study recruits in this work were patients ≥18 years with symptoms of suspected urinary tract infections, attending the outpatient’s clinics or admitted to the inpatient’s wards (within 48 hours of admission). Written informed consent was obtained from the patients prior to data collection. The methods were carried out in accordance with the relevant guidelines and regulations. All experimental protocols were approved by the Ethics Committee of the Faculty of Science, Beni-Suef University. Patients with history of antibiotics intake within the last 2 weeks were excluded.

### Sample size

Before the study, the number of required patients was determined after a power calculation according to data obtained in a previous study carried in Assiut, Egypt^44^. In that study the frequency of ESBL-producing *E. coli* was about 6.8%. A sample size of 80 patients in the group was determined to provide 80% power and 5% type I error with precision of 5.5% using the following equation:

$$\frac{{Z}_{1-\alpha /2}^{2}P(1-P)}{{d}^{2}}$$

*Z*_1−*α*/2_ = *Is standard normal variate* (*at 5% type 1 error* (*P* < 0.05) it is 1.96.

P = Expected proportion in population. d = Absolute error or precision.

### Isolation of *E. coli* from urine samples

A total of 583 midstream urine samples were collected by giving a sterile, dry, wide necked plastic container to every patient and were transported to the laboratory for processing within 2–4 h of collection. Positive cultures were identified by detection of at least 10^5^ CFU/ml^45^, after inoculation of a 10 μl (0.01 ml) of the urine sample into MacConkey agar (Oxoid, UK) and its incubation at 37 °C for 24 hours. *E. coli* identification was further done by inoculation on Eosin Methylene Blue (EMB) Agar plate and aerobic incubation at 37 °C for 24 hours. Gram staining of suspected colonies was performed (Gram negative bacilli). Citrate Utilization test was used to differentiate *E. coli* from other lactose-fermenters (Only negative with *E. coli*). *E. coli* strains were further confirmed by complete 16S rRNA detection by uniplex PCR.

### Phenotypic detection of ESBL production

All *E. coli* isolates were initially screened for ESBL production by inoculation on CHROMagar ESBL (CHROMagar, F-75006, Paris, France). All *E. coli* isolates, which showed dark pink to reddish color colonies on CHROMagar ESBL media were selected for further confirmation by the Modified Double Disc Synergy Test (MDDST)^46^. Briefly, isolates were inoculated on a plate containing a disc of amoxicillin-clavulanate (20/10 μg) at the center along with three 3^rd^ generation cephalosporins; cefotaxime, ceftriaxone, cefpodoxime and a 4^th^ generation cephalosporin; cefepime placed at 15 mm and 20 mm, respectively, centre to centre to that of the amoxicillin-clavulanate disc^47^. Any increase in the zone towards the disc of amoxicillin-clavulanate was considered as positive for ESBL production.

### Antibiotic susceptibility pattern of *E. coli* isolates

Antimicrobial susceptibility testing for phenotypically confirmed ESBL-isolates was determined by the disk diffusion method with reference to the standards of the Clinical and Laboratory Standards Institute^32^. The quality of antibiotic sensitivity was confirmed by using *E. coli* ATCC 25922 as a reference strain. Testing of antimicrobial susceptibilities of the following antibiotics was carried out: ampicillin (AMP 10 μg), piperacillin (PRL 100 μg), ofloxacin (OFX 5 μg), cefotaxime (CTX 30 μg), ceftazidime (CAZ 30 μg), cefpodoxime (CPD 10 μg), cefepime (FEP 30 μg), aztreonam (ATM), ampicillin/sulbactam (AMC 10/10 μg), sulphamethoxazole-trimethoprim (SXT 1.25/23.75 μg), chloramphenicol (C), gentamicin (CN 10 μg), ciprofloxacin (CIP 5 μg) and meropenem (MEM 10 μg) (Oxoid, UK). Multidrug resistance (MDR) was identified as having resistance to three or more classes of antibiotics.

### DNA extraction

Crude genomic bacterial DNA from all isolates with positive ESBL-screening results was extracted and purified using DNA extraction kits (Thermo Scientific, Gene JET Genomic, DNA Purification Kit, USA), according to manufacturer’s instructions.

### Phylogenetic grouping by quadruplex PCR

A quadruplex polymerase chain reaction (PCR)^12^ modified from the original triplex PCR method by Clermont and colleagues^48^ was used to group the *E. coli* isolates phylogenetically. This method was used to allocate the ESBL-producing *E. coli* isolates based on the presence or absence of 4 genes (arpA (400 bp), chuA (288 bp), yjaA (211 bp), TspE4.C2 (152 bp)) and allocating *E. coli* isolates into 1 of 8 phylo-groups (A, B1, B2, C, D, E, F and cryptic clade).

Multiplex PCR was carried out in a 25 μL reaction mixture, including 12.5 μl of MyTaq Red Mix (Bioline, USA Inc.), 1 μL (10 μM) each primer, 2.5 μL (nuclease free water), and 2 μL template DNA. Amplification was carried out as follows: initial denaturation at 94 C° (4 min), 30 cycles of denaturation at 94 °C (5 sec), annealing at 59 °C (10 sec) and elongation at 72 °C (10 sec), followed by final extension at 72 °C (5 min). The PCR products were analyzed electrophoretically by running the PCR product through 1% (w/v) agarose in Tris-borate-EDTA (TBE) at 90 V for 35 min and visualization under UV transillumination.

#### PCR amplification for 16s rrna, uniplex PCR for blatem, and duplex PCR for blashv and blactx-m amplification

Quality control of the DNA extraction was carried out by testing all extracted isolates for 16S rRNA by uniplex PCR^49^. This was followed by PCR detection of *ESBL* genes (*bla*_TEM_, *bla*_SHV_, *bla*_CTX-M_ genes) and subgrouping for *bla*_CTX-M_ genes into the 5 major groups (CTX-M-1(including CTX-M-15), CTX-M-2, CTX-M-8, CTX-M-9 and CTX-M-25), using primer pairs listed in Table 5 and amplification conditions as described previously^49–52^. The amplification reactions were carried out in 25 μl volumes containing 12.5 μl of MyTaq Red Mix (Bioline, USA Inc.), 1 μl of each primer (10 pmol/μl) and 2 μl of 100 ng/μl chromosomal DNA.

**Table 5:** Primers used in this work.

Gene	Primer (5′-3′)	size (bp)	ref.
16s rRNA	F-AGT TTG ATC MTG GCT CAG R-GGA CTA CHA GGG TAT CTA AT	797	^49^
*bla*_*TEM*_	F-ATG AGT ATT CAA CAT TTC CG R-CCA ATG CTT AAT CAG TGA GG	858	^50^
*bla*_*SHV*_	F-ATG CGT TAT ATT CGC CTG TG R-AGC GTT GCC AGT GCT CGA TC	862	^50^
Universal *bla*_CTX-M_	F-5′-SCS ATG TGC AGY ACC AGT AA R-5′-CCG CRA TAT GRT TGG TGG TG	554	^51,52^
*bla*_CTX-M-1_	F-5′-AAA AAT CAC TGC GCC AGT TC R-5′-AGC TTA TTC ATC GCC ACG TT	415	^51,52^
*bla*_CTX-M-2_	F-5′-CGA CGC TAC CCC TGC TAT T R-5′-CCA GCG TCA GAT TTT TCA GG	552	^51,52^
*bla*_CTX-M-8_	F- 5′-TCG CGT TAA GCG GAT GATGC R-5′-AAC CCA CGA TGT GGG TAG C	666	^51,52^
*bla*_CTX-M-9_	F-5′-CAA AGA GAG TGC AAC GGA R-TG 5′-ATT GGA GGT TCA TCA CC	205	^51,52^
*bla*_CTX-M-25_	F-5′-GCA CGA TGA CAT TCG GG R-5′-AAC CCA CGA TGT GGG TAG C	327	^51,52^
*bla*_CTX-M-15_	F-5′-ATAAAACCGGCAGCGGTG R-5′-GAATTTGACGATCGGGG	500	^51,52^
*chu*A	F-5′- GACGAACCAACGGTCAGGAT R-5′- TGCCGCCAGTACCAAAGACA	279	^12^
*yja*A	F-5′- TGAAGTGTCAGGAGACGCTG R-5′- ATGGAGAATGCGTTCCTCAAC	211	^12^
TspE4C2	F-5′- GAGTAATGTCGGGGCATTCA R-5′- CGCGCCAACAAAGTATTACG	152	^12^
*arp*A	5′-AACGCTATTCGCCAGCTTGC-3′ 5′-TCTCCCCATACCGTACGCTA-3′	400	^12^

### Statistical analysis

Analyses of data were performed by SPSS software (version 23) and proportions were compared using the Chi-square test (χ2 test) to determine the significant differences in resistance. Differences were considered significant at P < 0.05. Graphics were performed using Excel 2010. For description statistics, data are presented as mean standard deviation for continuous variables, as well as frequency and percentage for categorical variables.

## Data availability

Supplementary files are added.

## Supplementary information

Supplementary Information.
