# Supplementary material for: Molecular characterization of Extended-spectrum β lactamase- producing E. coli recovered from community-acquired urinary tract infections in Upper Egypt
Source: Sci Rep. 2020 Feb 17;10:2772. doi: 10.1038/s41598-020-59772-z (PMC7026060; doi:10.1038/s41598-020-59772-z)
Supplement: Supplementary file 1 — Supplementary Information. [file 41598_2020_59772_MOESM1_ESM.docx]

**Molecular characterization of Extended-spectrum β lactamase- producing *E. coli* recovered from community-acquired urinary tract infections in Upper Egypt**

Noha A Hassuna^1^*, Ahmed S Khairalla^2,3^, Eman M Farahat^4^, Adel M. Hammad^5^ and Medhat Abdel-Fattah^4^

**
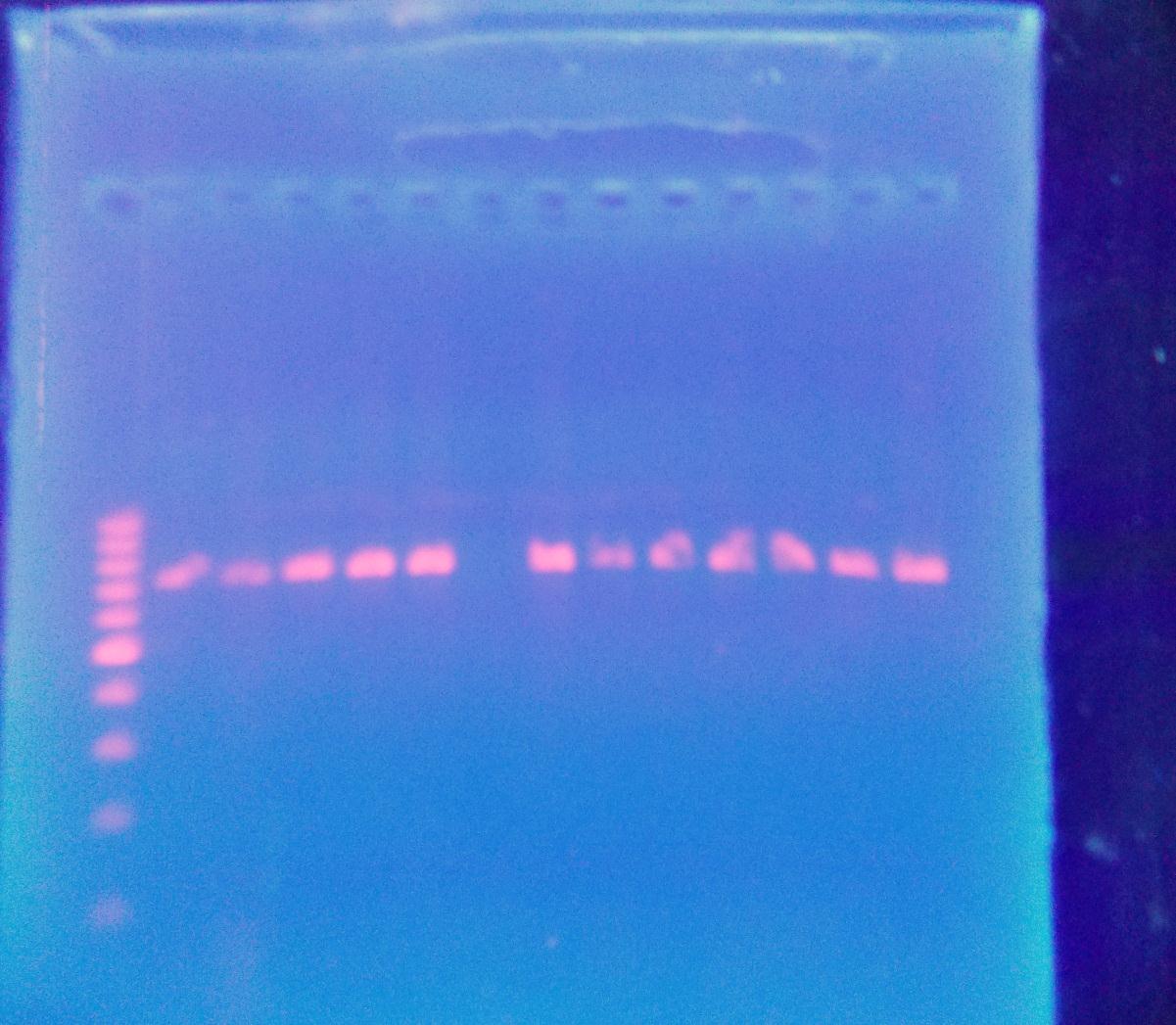
**

**Figure (S1): PCR amplification for 16SrRNA gene (797 bp).**

**
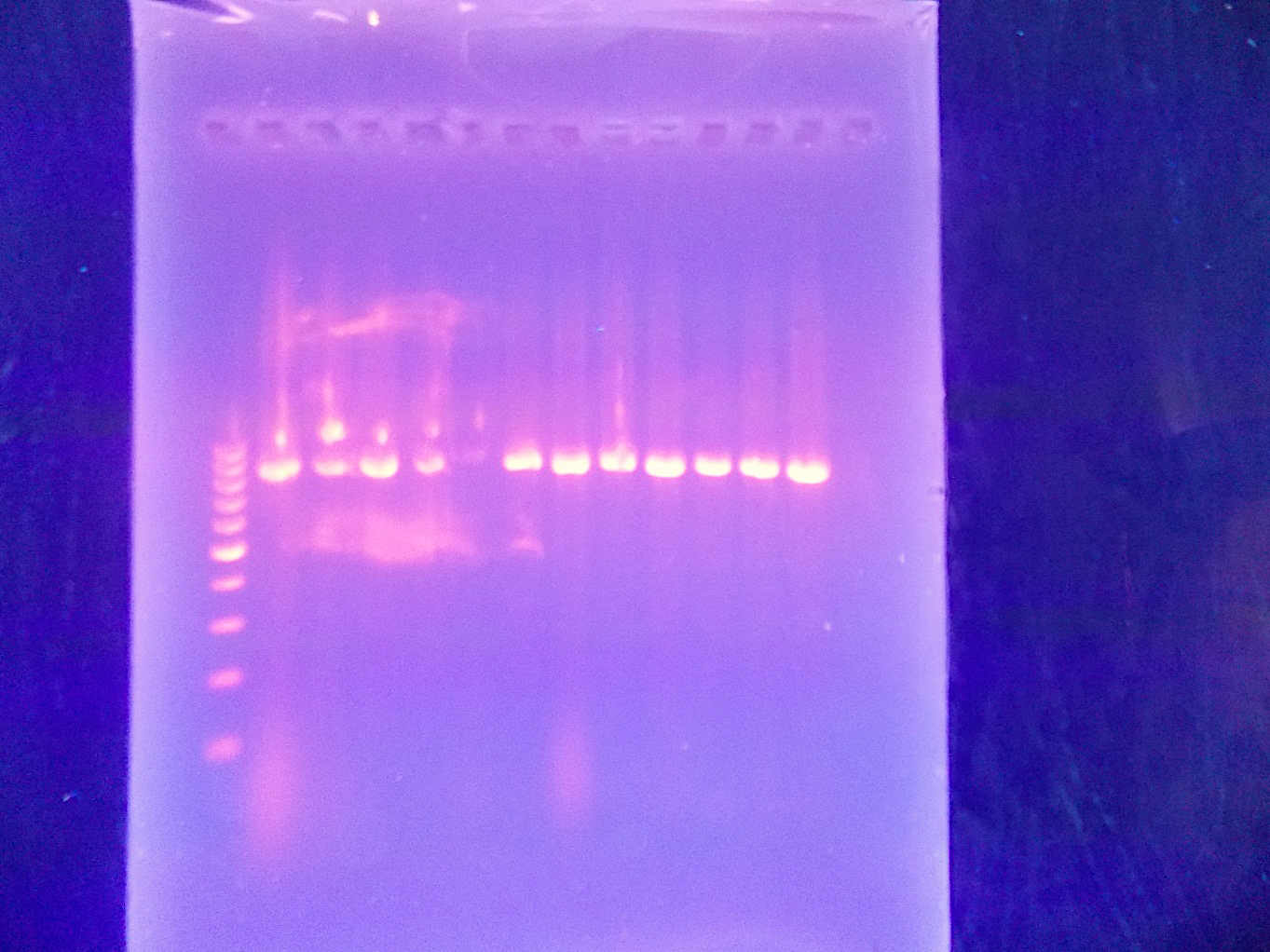
**

1000 bp

900 bp

800 bp

500 bp

**Figure (S2) PCR amplification for *bla*_TEM_ gene (858 bp).**

**
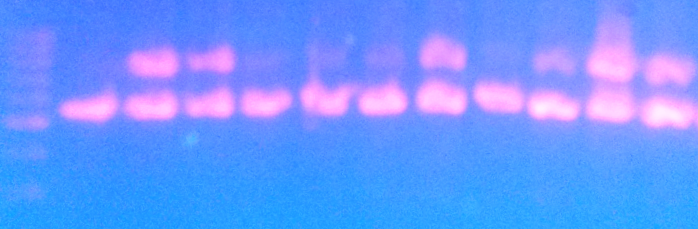
**

1000 bp

900 bp

800 bp

500 bp

**Figure (S3) PCR amplification *bla*_SHV_ (862 bp) and *bla*_CTX−M_(585 bp) genes**

**(CTX-M genes)**

**1 2 3 4 5 6 7 M**

**
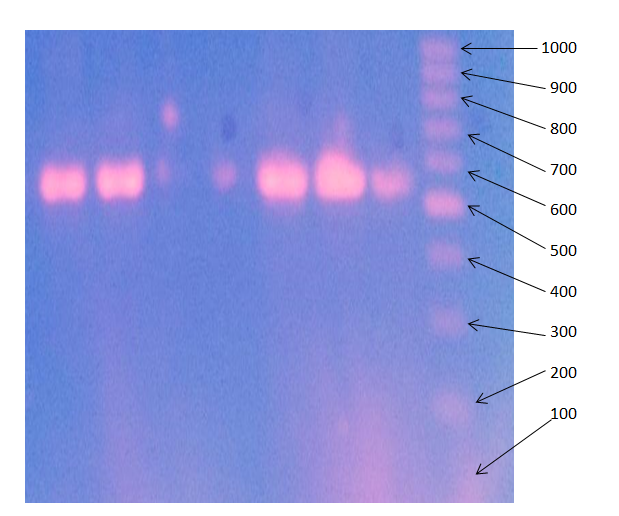
**

**Figure (S4): PCR amplification for *bla_CTX−M_*  Universal CTX-M (554bp). M: marker, 100–1000 bp DNA ladder.**

**M 1 2 3 4 5 6 7 8 9**

**600 bp**

**50**0 bp

400bp

300bp

200 bp

100 bp


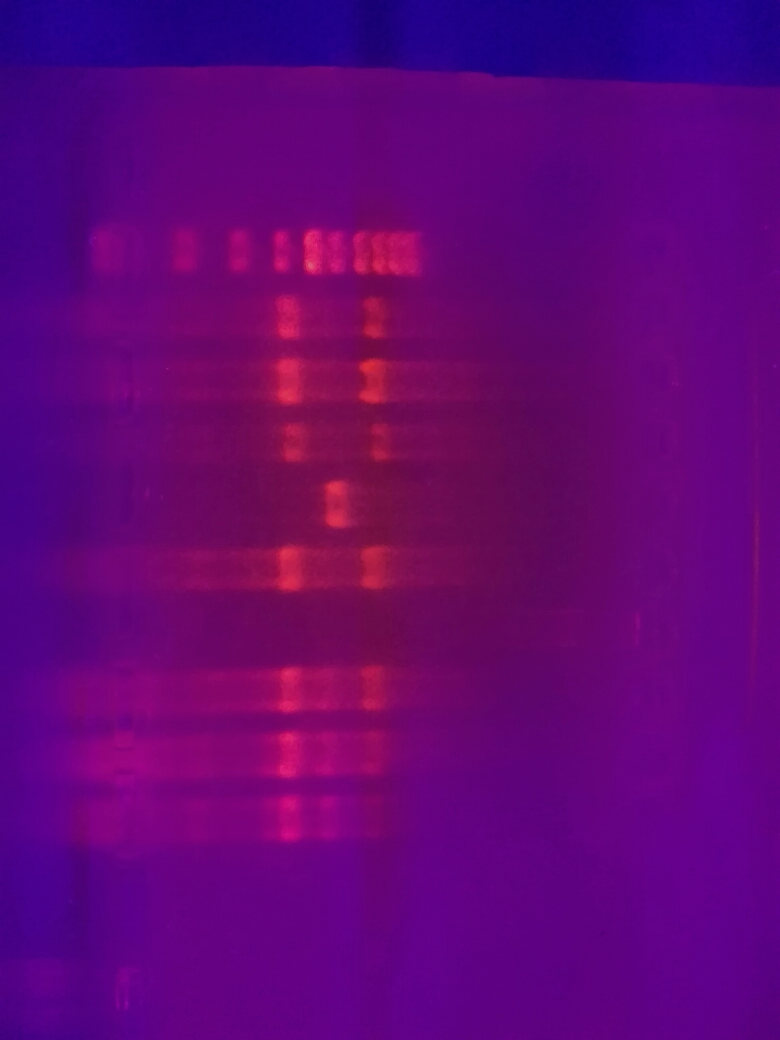


**Figure (S5): CTX-M-1 (415bp), CTX-M-8 (666bp) and CTX-M-2 (552bp) group enzymes in agarose gel electrophoresis. CTX-M-1 group in lane (1-2-3-5-7-8-9), CTX-M-8 group were found in lane (4) and CTX-M-2 group in lane (7-8-9). M: marker, 100–1000 bp DNA ladder.**


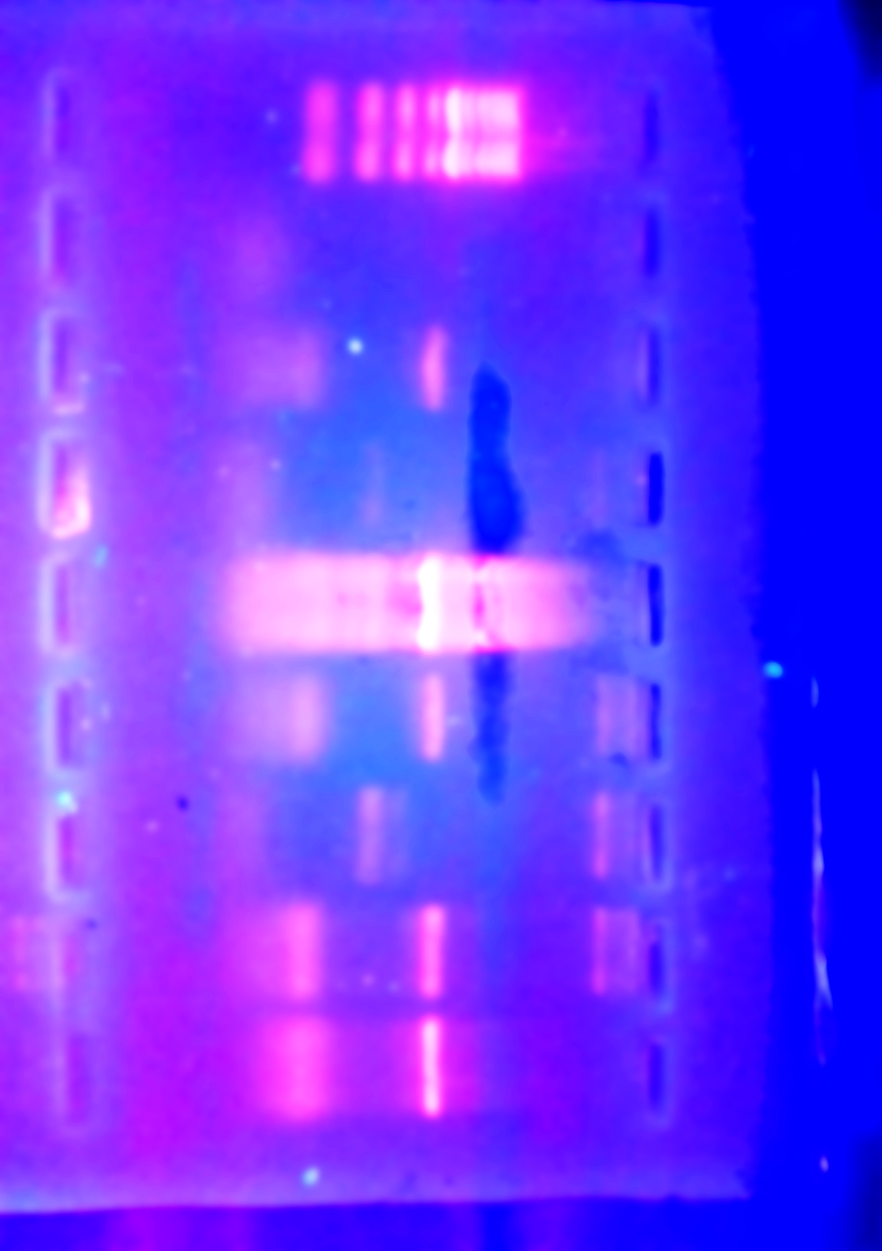
 **M 1 2 3 4 5 6 7 8**

**50**0 bp

400bp

300bp

200 bp

100 bp

**250**

**Figure (S6): CTX-M-1 (415bp) and CTX-M-9 (205bp) group enzymes in agarose gel electrophoresis. CTX-M-1 (415bp) group in lane (2-4-5-7-8) and CTX-M-9 group (205bp) in lane (6). M: marker, 100–1000 bp DNA ladder.**

**M 1 2 3 4 5 6**


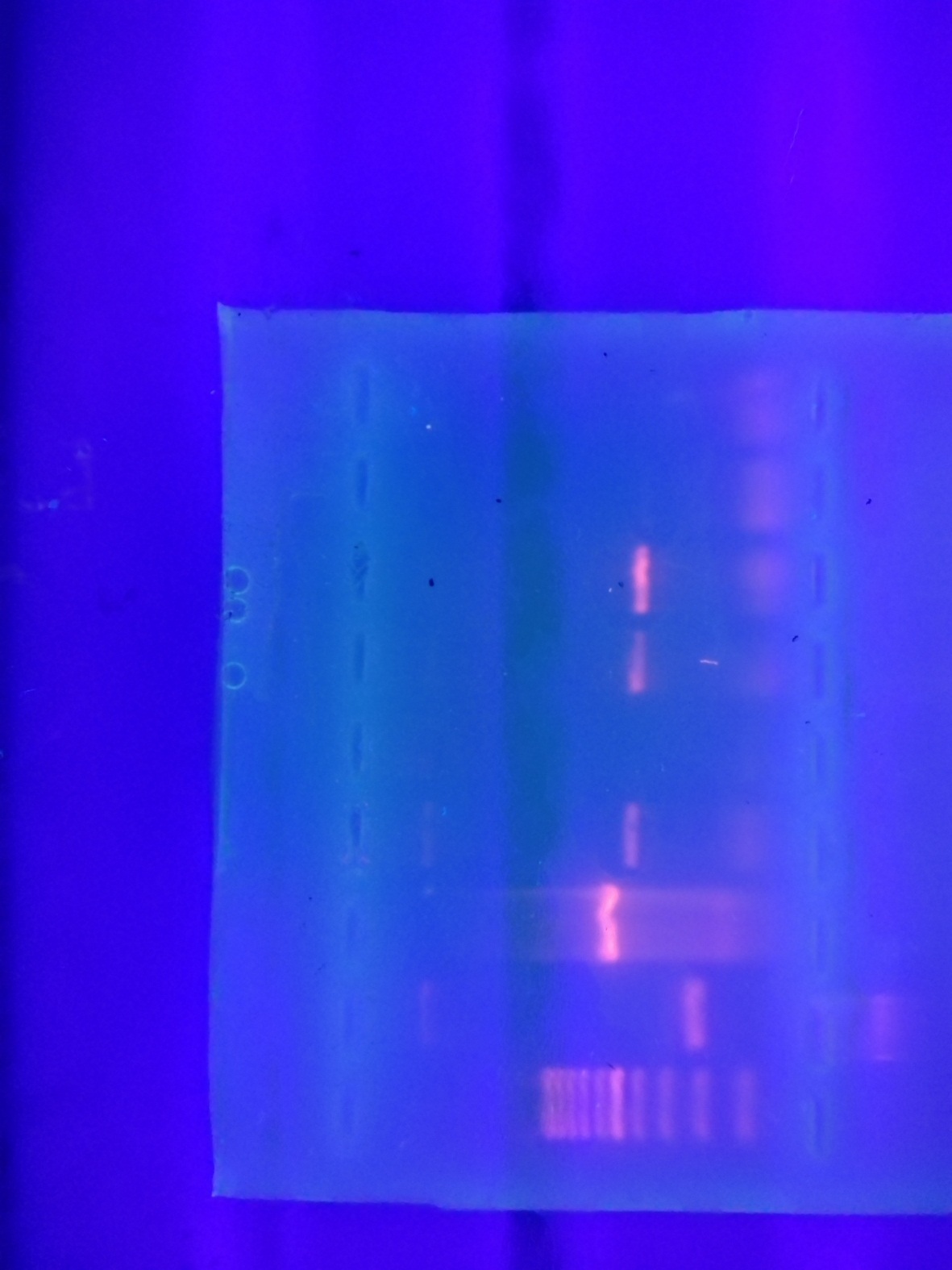


**Figure (S7): CTX-M-1 (415bp), CTX-M-9 (205bp) and CTX-M-15 (500bp) group enzymes in agarose gel electrophoresis. CTX-M-1 (415bp) group in lane (3-5-6), CTX-M-9 group (205bp) in lane (1) and CTX-M-15 (500bp) in lane (2). M: marker, 100–1000 bp DNA ladder.**
